# Galangin Activates Nrf2 Signaling and Attenuates Oxidative Damage, Inflammation, and Apoptosis in a Rat Model of Cyclophosphamide-Induced Hepatotoxicity

**DOI:** 10.3390/biom9080346

**Published:** 2019-08-05

**Authors:** Saleem H. Aladaileh, Mohammad H. Abukhalil, Sultan A. M. Saghir, Hamza Hanieh, Manal A. Alfwuaires, Amer A. Almaiman, May Bin-Jumah, Ayman M. Mahmoud

**Affiliations:** 1 Department of Medical Analysis, Princess Aisha Bint Al-Hussein Faculty of Nursing and Health Sciences, Al-Hussein Bin Talal University, Ma’an 71111, Jordan; 2 Department of Biology, Faculty of Science, Al-Hussein Bin Talal University, Ma’an 71111, Jordan; 3 Department of Biology, College of Science, King Faisal University, Al-Ahsa 31982, Saudi Arabia; 4 Department of Applied Medical Sciences, Community College of Unaizah, Qassim University, Buraydah 51431, Saudi Arabia; 5 Department of Biology, College of Science, Princess Nourah bint Abdulrahman University, Riyadh 84428, Saudi Arabia; 6 Physiology Division, Department of Zoology, Faculty of Science, Beni-Suef University, Beni-Suef 62511, Egypt

**Keywords:** galangin, cyclophosphamide, reactive oxygen species, nuclear factor erythroid 2-related factor 2, hepatotoxicity, inflammation

## Abstract

Cyclophosphamide (CP) is a widely used chemotherapeutic agent; however, its clinical application is limited because of its multi-organ toxicity. Galangin (Gal) is a bioactive flavonoid with promising biological activities. This study investigated the hepatoprotective effect of Gal in CP-induced rats. Rats received Gal (15, 30 and 60 mg/kg/day) for 15 days followed by a single dose of CP at day 16. Cyclophosphamide triggered liver injury characterized by elevated serum transaminases, alkaline phosphatase (ALP) and lactate dehydrogenase (LDH), and histopathological manifestations. Increased hepatic reactive oxygen species, malondialdehyde, nitric oxide, and oxidative DNA damage along with declined glutathione and antioxidant enzymes were demonstrated in CP-administered rats. CP provoked hepatic nuclear factor-kappaB (NF-κB) phosphorylation and increased mRNA abundance of inducible nitric oxide synthase (iNOS) and cyclooxygenase-2 (COX-2), and tumor necrosis factor-alpha (TNF-α) and interleukin-1 beta (IL-1β) both expression and serum levels. Gal prevented CP-induced liver injury, boosted antioxidants and suppressed oxidative stress, DNA damage, NF-κB phosphorylation and pro-inflammatory mediators. Gal diminished Bax and caspase-3, and increased B-cell lymphoma-2 (Bcl-2) in liver of CP-administered rats. In addition, Gal increased peroxisome proliferator-activated receptor gamma (PPARγ) expression and activated hepatic nuclear factor erythroid 2-related factor 2 (Nrf2) signaling showed by the increase in Nrf2, NAD(P)H: quinone acceptor oxidoreductase-1 (NQO-1) and heme oxygenase 1 (HO-1) in CP-administered rats. These findings suggest that Gal prevents CP hepatotoxicity through activation of Nrf2/HO-1 signaling and attenuation of oxidative damage, inflammation and cell death. Therefore, Gal might represent a promising adjuvant therapy to prevent hepatotoxicity in patients on CP treatment.

## 1. Introduction

Cyclophosphamide (CP) is an alkylating agent widely used for treating many human malignant tumors and as immunosuppressant drug [1,2,3]. However, its clinical application is often limited because of its adverse side effects, including hepatotoxicity [1,2,3]. Exposure to high doses of CP can induce acute hepatotoxic effects provoked by oxidative stress and activation of inflammatory cascade reaction [4,5]. The deleterious effects of CP are attributed to its metabolites phosphoramide mustard and acrolein produced through the action of hepatic microsomal cytochrome P450 (CYP450) [6]. The highly reactive metabolite acrolein has a short biological half-life and triggers the production of reactive oxygen species (ROS) [7]. In turn, ROS provoke lipid peroxidation (LPO), protein carbonylation and oxidative DNA damage [5,8], and activate multiple signaling molecules, including nuclear factor-kappaB (NF-κB) [1,4], eventually resulting in cell death. Oxidative stress and inflammation can activate the apoptotic signaling pathways in hepatocytes [5] and are therefore implicated in CP hepatotoxicity.

The cells are equipped with multiple defense mechanisms to counteract ROS-induced oxidative damage. These defenses include several well-coordinated antioxidant enzymes that neutralize excess ROS. Several cytodefensive proteins are modulated by the nuclear factor erythroid 2-related factor 2 (Nrf2), a central regulator of inducible antioxidant enzymes conferring protection against excess ROS [9]. Under basal conditions, Nrf2 is sequestered in the cytoplasm by Kelch-like ECH-associated protein 1 (Keap1) which serves as a sensor protein against electrophiles and ROS. Upon exposure to moderate oxidative stress, Nrf2 is released and translocate into the nucleus where it binds to the antioxidant response element (ARE) in the DNA promoter region and initiates the transcription of heme oxygenase (HO)-1, NAD(P)H: quinone oxidoreductase 1 (NQO-1), superoxide dismutase (SOD), and catalase (CAT), and other antioxidant genes [9]. Activation of Nrf2 signaling has evinced protection against oxidative damage induced by drugs and other agents [9,10,11]. In contrast, the lack of Nrf2 increased the severity of acetaminophen-induced hepatotoxicity in mice [12]. These findings demonstrated the crucial role of Nrf2 in preventing drug toxicity mainly through boosting cellular antioxidant defenses. Moreover, activation of peroxisome proliferator activated receptor gamma (PPARγ) has been reported to protect against liver injury, fibrogenesis, and carcinogenesis in rodents [1,13,14,15,16]. PPARγ is a ligand-activated regulator of lipid and carbohydrate metabolism, and cell proliferation and differentiation [17]. Its activation induces anti-inflammatory responses and directly modulates the expression of several antioxidant genes [18]. Thus, activation of PPARγ might represent an effective strategy to prevent oxidative stress and inflammation associated with chemotherapeutic agents.

Galangin (Gal, 3,5,7-trihydroxyflavone) is a bioactive flavonoid found in honey and *Alpinia officinarum* and possesses promising therapeutic properties [19]. Gal was shown to be safe and well tolerated without biological adverse effects in rodents [20]. Preclinical investigations have demonstrated the antioxidant and anti-inflammatory efficacies of Gal in diabetes, nephrotoxicity, and fructose-fed animal models [20,21,22]. In addition, Gal protected against carbon tetrachloride (CCl_4_)-induced hepatotoxicity and fibrosis by mitigating oxidative stress and inhibiting hepatic stellate cells activation and proliferation [23]. Gal was also found to reduce apoptosis by restoration of antioxidant defense mechanisms in ischemia-reperfusion (I/R)-induced liver injury in rats [24]. Another study showed that Gal can protect human keratinocytes against ultraviolet B-induced oxidative damage [25]. In a recent study, Gal has been proven to attenuate liver injury in diabetic rats through maintaining liver mitochondrial function and decreasing oxidative stress [26]. Although several pharmacological actions of Gal have been investigated, nothing is known about its potency to prevent CP-induced liver injury. We investigated the potential of Gal to prevent CP hepatotoxicity, emphasizing on its effect on oxidative damage, inflammation, and cell death, and the possible involvement of PPARγ and Nrf2/HO-1 signaling.

## 2. Materials and Methods 

### 2.1. Animals and Experimental Design

Thirty-six male Wistar rats (180–200 g) obtained from VACSERA (Giza, Egypt) were used in this study. The rats were housed in standard cages at normal temperature (23 ± 1 °C) on a 12 h light/dark cycle and received a standard chow diet and water *ad libitum*. The protocol and procedures were approved by the local animal care review committee and performed in line with the guidelines of the National Institutes of Health (NIH publication No. 85–23, revised 2011). After acclimatization for one week, the rats were divided into six groups (*N* = 6) as following:

Group I: Control.

Group II: Received 60 mg/kg Gal for 15 days.

Group III: Received CP (150 mg/kg) at day 16 [13].

Group IV: Received 15 mg/kg Gal for 15 days and CP (150 mg/kg) at day 16.

Group V: Received 30 mg/kg Gal for 15 days and CP (150 mg/kg) at day 16.

Group VI: Received 60 mg/kg Gal for 15 days and CP (150 mg/kg) at day 16.

Cyclophosphamide (Endoxan^®^) is a product of Baxter Oncology GmbH (Halle, Germany), dissolved in physiological saline, and administered intraperitoneally (i.p.). Galangin was supplied by Nanjing Zelang Med. Tech. Inc. (Nanjing, China), dissolved in 0.5% carboxymethyl cellulose (CMC), and administered via oral gavage. The doses of Gal were chosen according to previous reports demonstrated its in vivo effects at 8–100 mg/kg [20,21,23]. Group I and III received 0.5% CMC orally for two weeks, and groups I and II received an i.p. injection of physiological saline at day 16. Three days after CP injection (day 19), all groups were anaesthetized, and blood samples were collected by cardiac puncture. The livers were removed and washed with cold phosphate buffered saline (PBS). Liver specimens from different groups were fixed in 10% neutral buffered formalin (NBF) for histopathological examination and other samples were homogenized in PBS (10% *w/v*), whereas others were stored at −80 °C.

### 2.2. Determination of Liver Function Markers and Cytokines

Serum alanine aminotransferase (ALT), aspartate aminotransferase (AST), alkaline phosphatase (ALP), and lactate dehydrogenase (LDH) were assayed using Spinreact (Girona, Spain) Kits. Serum tumor necrosis factor alpha (TNF-α) and interleukin-1 beta (IL-1β) were determined using ELISA kits (R&D Systems, Minneapolis, MN, USA). All assays were performed following the manufacturers’ instructions.

### 2.3. Determination of Reactive Oxidative Species, Lipid Peroxidation, Nitric Oxide, and Antioxidants

Liver homogenates were centrifuged, and the clear supernatants were used for the assays. ROS were immediately determined using 2′,7′-dichlorodihydrofluorescein diacetate [11], and malondialdehyde (MDA) [27], nitric oxide (NO) [28], reduced glutathione (GSH) [29], glutathione disulfide (GSSG) [30], SOD [31], CAT [32], and glutathione peroxidase (GPx) [33] were assayed in the liver homogenate samples.

### 2.4. Determination of 8-Oxo-2′-Deoxyguanosine (8-Oxo-dG) and Caspase-3

The levels of 8-Oxo-dG and caspase-3 were assayed in the liver homogenates by ELISA kits supplied by Cusabio (Wuhan, China).

### 2.5. Histological Preparation of Liver Sections

Specimens from liver were fixed 10% NBF for 24 h and processed for paraffin embedding. After cutting, the 5 μm sections were stained with hematoxylin and eosin (H&E), and examined using a light microscope.

### 2.6. Gene Expression

The messenger RNA (mRNA) expression levels of cyclooxygenase-2 (COX-2), inducible nitric oxide synthase (iNOS), TNF-α, IL-1β, BAX, BCL-2, NRF2, HO-1, NQO-1, PPARγ, caspase-3, and CYP450s were quantified using quantitative reverse transcription-polymerase chain reaction (qRT-PCR), as previously described [34]. Total RNA was extracted using TRIzol (Invitrogen, Waltham, MA, USA), treated with RNase-free DNase (Qiagen, Düsseldorf, Germany), quantified, and its quality was examined using formaldehyde-agarose electrophoresis. Samples with OD260/OD280 nm absorption ratio ≥ 1.8 were used for cDNA synthesis which has been amplified using Qiagen SYBR green master mix, and the set of primers is listed in Table 1. The obtained amplification data were analyzed by the 2^−ΔΔCt^ method [35], using β-actin as a house-keeping gene.

### 2.7. Western Blotting

Immunoblot analysis of NF-κB p65 and Nrf2 in the liver tissue was performed as described earlier [1]. Liver tissues were homogenized in ice-cold radioimmunoprecipitation assay (RIPA) buffer with proteinase and phosphatase inhibitors. Total protein content in the homogenates was assayed using Bradford reagent and 40 µg proteins were separated under denaturing conditions using 10% sodium dodecyl sulfate-polyacrylamide gel electrophoresis (SDS-PAGE) and electrophoretically transferred to nitrocellulose membranes. The membranes were blocked and probed with anti-Nrf2, anti-NF-κB p65, and anti-β-actin (Novus Biologicals, Centennial, CO, USA) primary antibodies overnight at 4 °C. The membranes were washed three times and incubated with appropriate secondary antibodies (Novus Biologicals) and the blots were developed using enhanced chemiluminescence detection kit (BIO-RAD, Hercules, CA, USA). The band intensity was quantified using ImageJ.

### 2.8. Statistical Analysis

Results are expressed as mean ± standard error of the mean (SEM). All statistical comparisons among groups were determined by one-way ANOVA followed by Tukey’s post-hoc test for multiple comparisons using GraphPad Prism 7 software (San Diego, CA, USA). A *P* value < 0.05 was considered significant.

## 3. Results

### 3.1. Galangin Protects against CP-Induced Hepatic Injury

**Figure 1:**
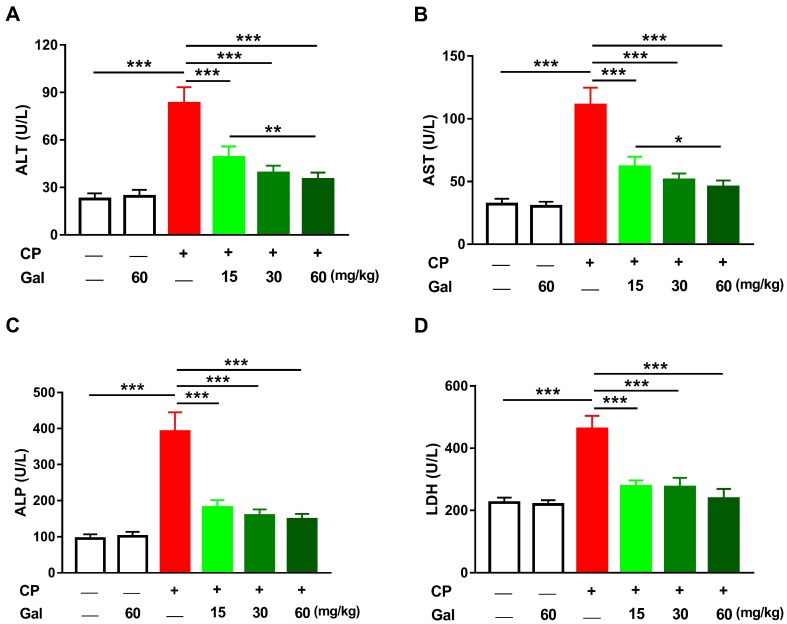
Galangin (Gal) prevents liver injury in cyclophosphamide (CP)-intoxicated rats. Gal ameliorated (**A**) alanine aminotransferase (ALT), (**B**) aspartate aminotransferase (AST), (**C**) alkaline phosphatase (ALP), and (**D**) lactate dehydrogenase (LDH) in serum of rats received CP. Data are mean ± SEM, (*N* = 6). * *P* < 0.05, ** *P* < 0.01, and *** *P* < 0.001.

**Figure 2:**
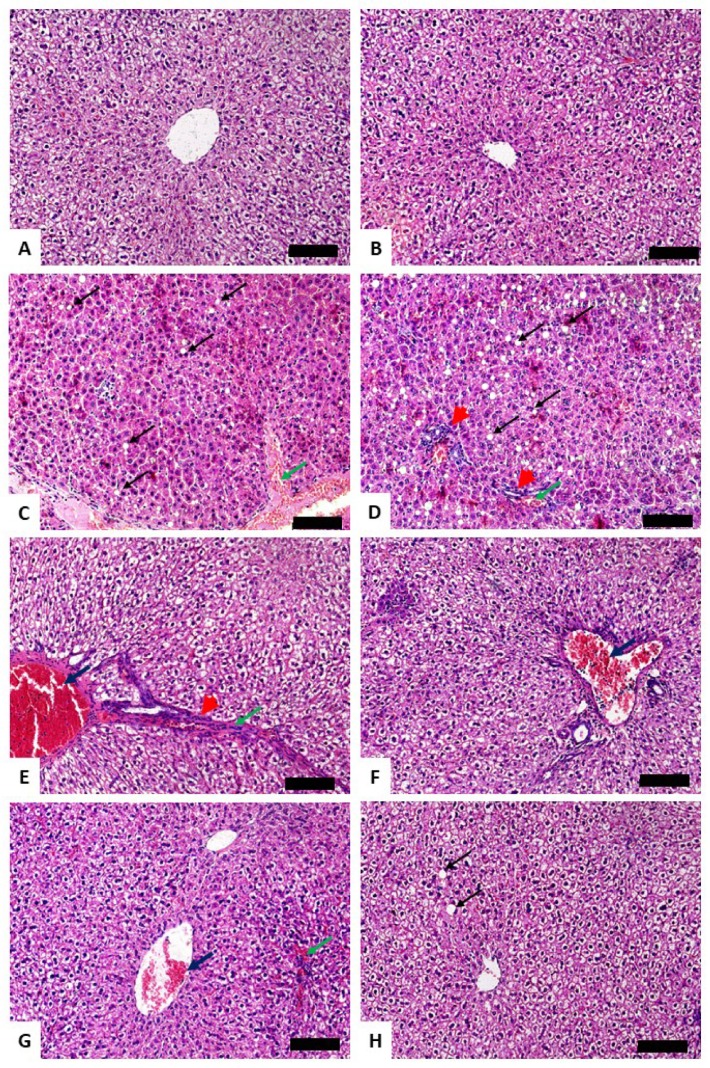
Photomicrographs of liver sections of (**A**) control and (**B**) Gal-treated rats showing normal liver architecture, (**C**–**E**) CP-intoxicated rats showing degenerative changes and cytoplasmic vacuolations (black arrow), leukocyte infiltration (arrowhead), hemorrhage (green arrow), and congestions (blue arrow), and CP-induced rats pre-treated with (**F**) 15 mg/kg, (**G**) 30 mg/kg, and (**H**) 60 mg/kg Gal showing mild congestion (blue arrow), hemorrhage (green arrow), and cytoplasmic vacuolations (black arrow). (Hematoxylin and eosin (H&E); X200) (Scale bar = 100 µm).

Serum transaminases, ALP and LDH, were assayed to assess the preventive effect of Gal on CP hepatotoxicity. ALT, AST, ALP, and LDH were elevated significantly in CP-induced rats (*P* < 0.001) as depicted in Figure 1A–D. Pre-treatment with Gal (15, 30, and 60 mg/kg) ameliorated all assayed liver function markers in CP-induced rats. Gal showed a dose-dependent ameliorative effect on serum ALT and AST levels. Oral supplementation of 60 mg/kg Gal did not alter liver function markers in normal animals (Figure 1A–D). Similarly, Gal had no effect on the histological architecture of the liver in normal animals. Examination of the H&E-stained sections showed normal liver architecture in control (Figure 2A) and Gal-supplemented rats (Figure 2B). The CP-intoxicated rats showed degenerative changes, leukocyte infiltration, hemorrhage, cytoplasmic vacuolations, congestions, and other manifestations (Figure 2C–E and Table 2). In contrast, rats received Gal (15 (Figure 2F), 30 (Figure 2G), and 60 mg/kg (Figure 2H)) before CP injection exhibited remarkable amelioration of the liver histology with mild cytoplasmic vacuolations, leukocyte infiltration, and congestion (Table 2).

### 3.2. Galangin Prevents CP-Induced Alterations in the Expression of CYPs

**Figure 3:**
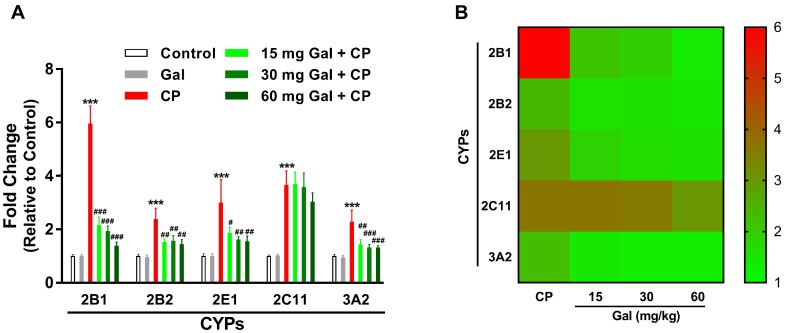
Galangin (Gal) prevents CP-induced alterations in the expression of cytochromes (CYPs). (**A**) Gal down-regulated CYPs 2B1, 2B2, 2E1, and 3A2 mRNA abundance in CP-induced rats. Data are mean ± SEM, (*N* = 6). *** *P* < 0.001 versus Control, and ^#^ *P* < 0.05, ^##^ *P* < 0.01, and ^###^ *P* < 0.001 versus CP. (**B**) Heat map showing the effect of Gal on the expression of CYPs in CP-induced rats.

The gene expression data showed the non-significant effect of Gal on the expression levels of CYPs 2B1, 2B2, 2E1, 2C11, and 3A2 in liver of normal rats (Figure 3). CP triggered a significant up-regulation of CYPs 2B1, 2B2, 2E1, and 3A2 mRNA abundance, an effect that was significantly inhibited by Gal. The CYP2C11 mRNA was significantly increased in the liver of both control and Gal-pre-treated CP-induced rats as represented in Figure 3. 

### 3.3. Galangin Attenuates Oxidative Stress and DNA Damage, and Enhances Antioxidants in Liver of CP-Induced Rats

**Figure 4:**
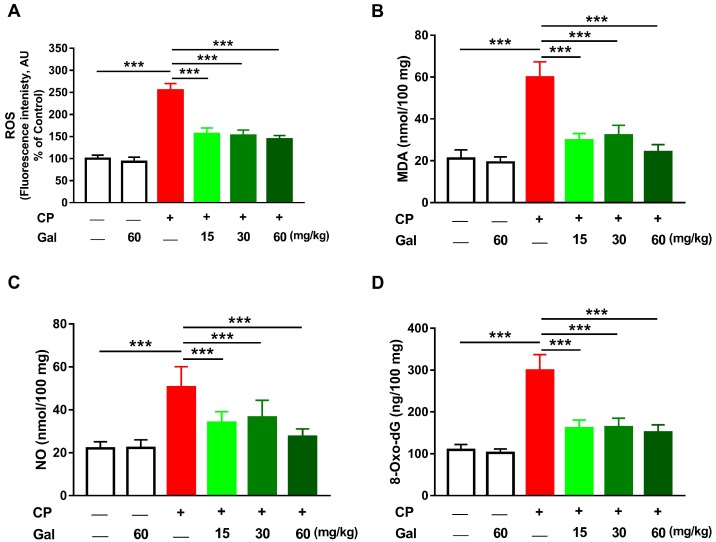
Galangin (Gal) suppresses oxidative stress in CP-intoxicated rats. Gal suppressed hepatic (**A**) reactive oxygen species (ROS), (**B**) malondialdehyde (MDA), (**C**) nitric oxide (NO), and (**D**) 8-Oxo-dG. Data are mean ± SEM, (*N* = 6). *** *P* < 0.001.

**Figure 5:**
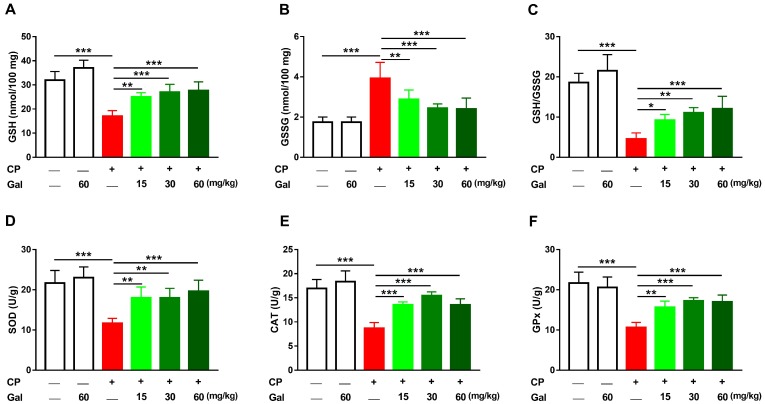
Galangin (Gal) enhances cellular antioxidants in CP-intoxicated rats. Gal increased (**A**) Reduced glutathione (GSH), (**C**) GSH/glutathione disulfide (GSSG) ratio, and activity of (**D**) superoxide dismutase (SOD), (**E**) catalase (CAT), and (**F**) glutathione peroxidase (GPx), and decreased GSSG (**B**) in the liver of CP-intoxicated rats. Data are mean ± SEM, (*N* = 6). * *P* < 0.05, ** *P* < 0.01, and *** *P* < 0.001.

Since CP toxicity is associated with oxidative stress, this study has evaluated the effect of Gal on ROS, LPO, and NO. CP triggered a remarkable (*P* < 0.001) increase in hepatic ROS (Figure 4A), LPO (Figure 4B), and NO (Figure 4C) levels. CP-induced oxidative stress resulted in increased hepatic 8-Oxo-dG levels (Figure 4D). Pre-treatment with different doses of Gal prevented oxidative damage in CP-intoxicated rats, with no effect on the liver of normal animals. Rats treated with 60 mg/kg Gal showed normal GSH (Figure 5A) and antioxidant enzymes (Figure 5B–D). CP decreased hepatic GSH (Figure 5A), GSH/GSSG ratio (Figure 5C), SOD (Figure 5D), CAT (Figure 5E), and GPx (Figure 5F), and increased GSSG levels (Figure 5B); an effect prevented by all doses of Gal.

### 3.4. Galangin Suppresses NF-κB and Inflammation in CP-Intoxicated Rats

**Figure 6:**
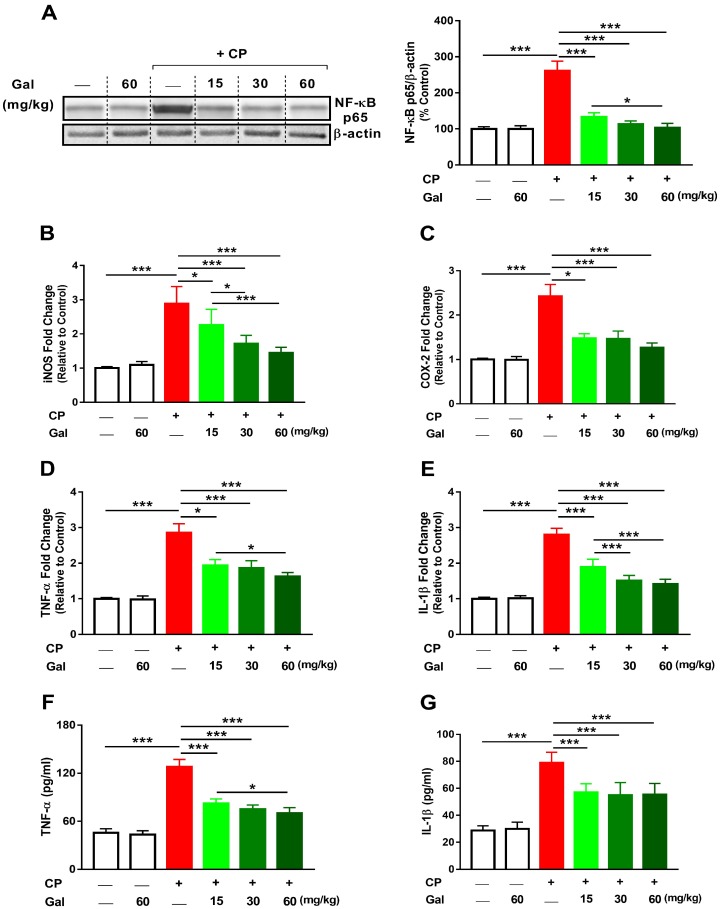
Galangin (Gal) suppresses inflammation in CP-intoxicated rats. Gal reduced hepatic (**A**) NF-κB p65, mRNA abundance of (**B**) iNOS, (**C**) COX-2, (**D**) TNF-α, and (**E**) IL-1β, and serum levels of (**F**) TNF-α and (**G**) IL-1β. Data are mean ± SEM, (*N* = 6). * *P* < 0.05 and *** *P* < 0.001.

The effect of Gal on inflammation was explored through the determination of NF-κB phosphorylation, mRNA abundance of inflammatory mediators, and serum pro-inflammatory cytokines. CP provoked inflammation marked by increased hepatic NF-κB p65 (*P* < 0.001; Figure 6A), and gene expression of iNOS, COX-2, TNF-α and IL-1β (Figure 6B–E). In addition, TNF-α (Figure 6F) and IL-1β (Figure 6G) were elevated significantly (*P* < 0.001) in serum of CP-induced animals. All doses of Gal administered before CP effectively suppressed NF-κB phosphorylation and expression of pro-inflammatory mediators as well as serum TNF-α and IL-1β. Gal exerted a dose-dependent effect on NF-κB p65, iNOS, TNF-α (mRNA and serum levels), and IL-1β mRNA. All of the assayed inflammation markers were not affected in normal rats received 60 mg/kg Gal.

### 3.5. Galangin Attenuates CP-Induced Hepatic Apoptosis

**Figure 7:**
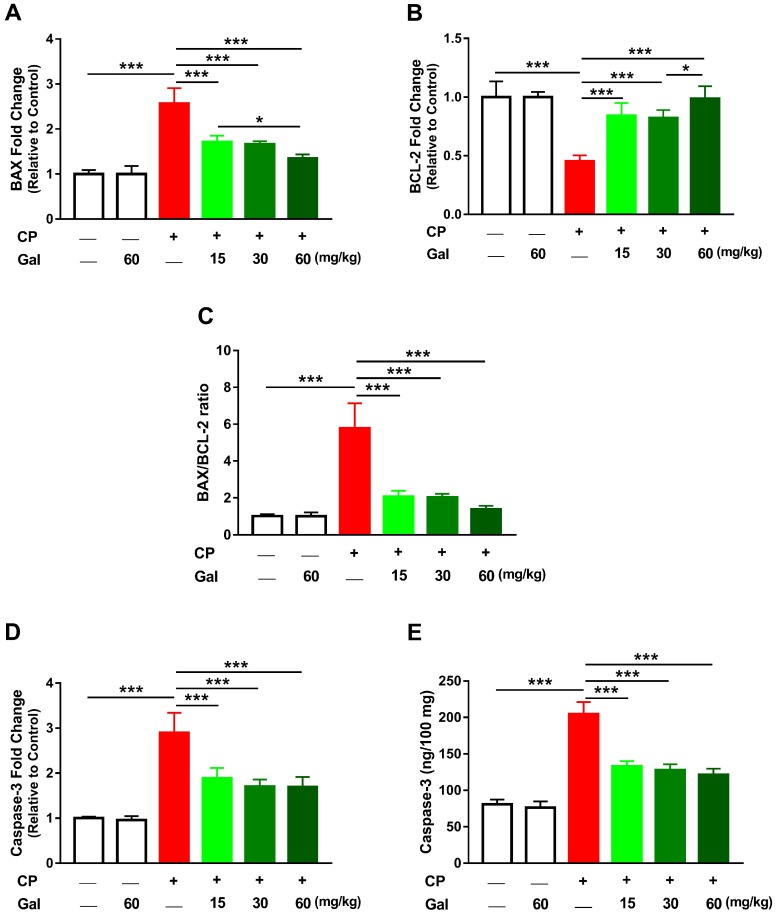
Galangin (Gal) attenuates CP-induced hepatic apoptosis in CP-intoxicated rats. Gal increased BCL-2 (**B**) and decreased (**A**) BAX, (**C**) BAX/BCL-2 ratio, (**D**) caspase-3 mRNA, and (**E**) caspase-3 activity. Data are mean ± SEM, (*N* = 6). * *P* < 0.05 and *** *P* < 0.001.

CP augmented hepatic mRNA expression of BAX (*P* < 0.001; Figure 7A) coupled with a significant decrease in BCL-2 (Figure 7B). The ratio of BAX/BCL-2, caspase-3 mRNA, and caspase-3 activity (Figure 7C-E) were boosted in the liver of CP-injected rats (*P* < 0.001). Pre-treatment with Gal (15, 30, and 60 mg/kg) resulted in a dose-dependent decrease in BAX and increased BCL-2 in CP-induced rats. The anti-apoptotic effect of Gal was supported by the significantly reduced BAX/BCL-2 ratio and caspase-3 both mRNA and activity. All markers of apoptosis showed non-significant changes in liver of normal rats treated with Gal (Figure 7A–E).

### 3.6. Galangin Activates Nrf2/HO-1 Signaling and Increases PPARγ Expression Liver of CP-Intoxicated Rats

**Figure 8:**
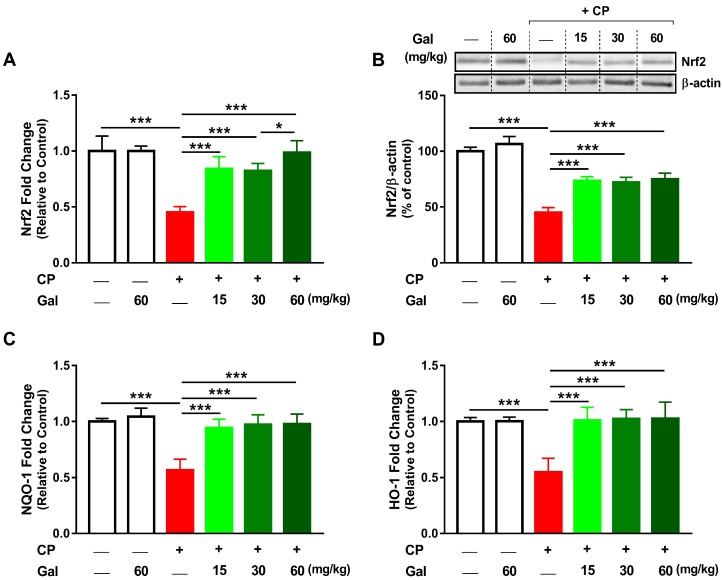
Galangin (Gal) activates Nrf2/HO-1 signaling in CP-intoxicated rats. Gal increased (**A**) Nrf2 mRNA, (**B**) Nrf2 protein, (**C**) NQO-1 mRNA, and (**D**) HO-1 mRNA in liver of CP-induced rats. Data are mean ± SEM, (*N* = 6). * *P* < 0.05 and *** *P* < 0.001.

CP diminished hepatic Nrf2 both mRNA (Figure 8A) and protein expression (Figure 8B) in rats (*P* < 0.001). The suppressed Nrf2 signaling in CP-intoxicated rats was confirmed by reduced NQO-1 (Figure 8C) and HO-1 (Figure 8D) gene expression (*P* < 0.001). Rats received Gal (15, 30, or 60 mg/kg) before CP showed remarkable alleviation in hepatic levels of Nrf2, NQO-1, and HO-1. While the effect of Gal on Nrf2 mRNA abundance was dose-dependent, non-significant differences between different doses were observed with regards to Nrf2 protein, NQO-1, and HO-1 mRNA.

**Figure 9:**
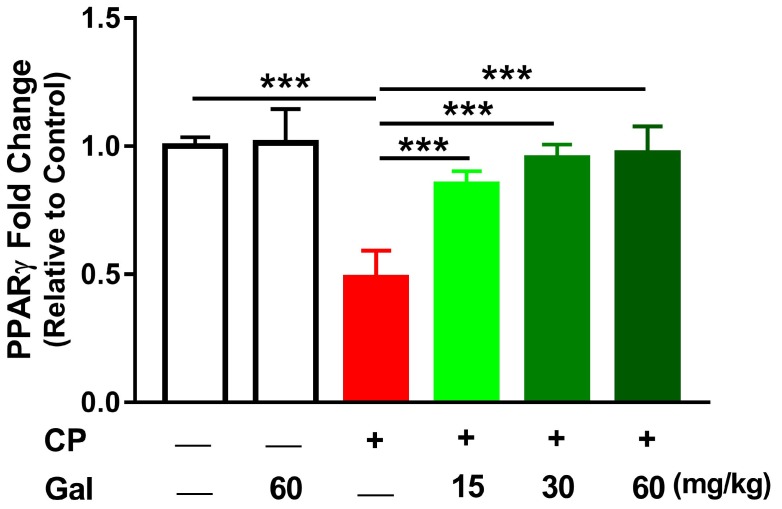
Galangin (Gal) increases peroxisome proliferator activated receptor gamma (PPARγ) expression in liver of CP-intoxicated rats. Data are mean ± SEM, (*N* = 6). *** *P* < 0.001.

We also investigated the effect of Gal on hepatic PPARγ mRNA as depicted in Figure 9. CP suppressed hepatic PPARγ mRNA (*P* < 0.001), whereas all doses of Gal prevented the suppressive effect of CP on hepatic PPARγ (*P* < 0.001). Of note, Gal alone affected neither hepatic Nrf2/HO-1 signaling (Figure 8) nor PPARγ (Figure 9) in normal rats.

## 4. Discussion

Hepatotoxicity is a serious side effect that limits the use of CP in treatment. CP has been documented to trigger oxidative stress-mediated liver toxicity and injury [1,13]. Although CP hepatotoxicity has been a research focus of many researchers, the currently preventive strategies are still very limited and there is a need to develop novel approaches to prevent chemotherapy-induced organ injury. Herein, we investigated the efficacy of Gal, a natural flavonoid with promising therapeutic effects, to prevent CP hepatotoxicity in rats. Our results showed that Gal can attenuate liver injury by up-regulating Nrf2/HO-1 signaling and PPARγ, resulting in the suppression of oxidative stress, DNA damage, inflammation, and cell death in CP-induced rats.

Consistent with several previous studies [1,4,13], CP hepatotoxicity was evidenced by elevated transaminases and other liver function markers along with histopathological manifestations, including degenerative changes, vacuolations, inflammatory cell infiltration, and others. Transaminases, ALP and LDH, are commonly used as indicators of liver function and sensitive markers of hepatocellular degenerative and necrotic changes [36]. Previous studies have described the elevation of serum transaminases and histological alterations as the main consequences of CP [1,34,37]. Pre-treatment with different doses of Gal prevented liver injury and ameliorated liver function in CP-induced rats, demonstrating a potent hepatoprotective efficacy. In accordance, Gal ameliorated serum aminotransferases and maintained normal architectural integrity of hepatocytes in murine models of I/R injury [24], concanavalin A-induced hepatitis [38], and CCl_4_ hepatotoxicity [23].

CYP2 and CYP3 are particularly important for drug metabolism [39]; therefore, we studied the effect of Gal on the expression levels of CYPs 2B1, 2B2, 2E1, 2C11, and 3A2 in liver of normal and CP-induced rats. Although oral administration of Gal did not induce changes in the expression levels of all assayed genes in liver of normal rats, CP-induced rats exhibited a significant increase in the mRNA abundance of CYPs 2B1, 2B2, 2E1, 2C11, and 3A2. Previous studies have reported increased expression of CYPs 2E1, 3A4, 2B1, and 2B2 in the liver of mice received repeated administration of CP [40]. CYPs 2B1, 2B2, 2C11, and 3A2 were up-regulated in rats received a single intravenous dose of either 40 or 200 mg/kg CP [41]. All doses of Gal reduced the expression levels of CYPs 2B1, 2B2, 2E1, and 3A2 in CP-induced rats, whereas exerted no effect on CYP2C11. Therefore, modulation of CYPs by Gal can decrease the formation of toxic metabolites and liver injury in CP-administered rats. This notion is supported by the study of Sheweita et al. [40] who demonstrated that the inhibition of CYPs ameliorated liver injury in CP-induced mice. Owing to the ability of flavonoids to modulate the aromatic hydrocarbon receptor (AhR) [42], pregnane X receptor (PXR) [43], and constitutive androstane receptor (CAR) [44], Gal has been speculated to exert its effect on the expression of CYPs by modulating these nuclear receptors [45].

The antioxidant efficacy of Gal has been reported in diabetes, nephrotoxicity, and fructose-fed animals [20,21,22]. Therefore, we assumed the hepatoprotective effect of Gal to be mediated, at least in part, via its antioxidant efficacy. Increased ROS and oxidative damage represent the main culprit behind the toxicity of CP. Acrolein, released during the metabolism of CP, can provoke membrane damage by direct covalent binding to lipids and proteins and formation of free reactive radicals [7]. CP-mediated elevation of ROS can cause marked damage in cells through LPO, protein oxidation, depletion of antioxidants, and DNA damage. LPO can disrupt membrane fluidity and permeability and inactivate membrane-bound proteins, eventually leading to destruction of the membrane [46]. Moreover, free radicals may disrupt structural proteins conformation and alter the active sites of defensive enzymes, raising havoc throughout the cell [46]. Here, CP provoked an increase in ROS and LPO, and depleted GSH and antioxidant enzymes in rat liver. These findings added support to our previous studies where LPO and diminished antioxidants were reported in CP-induced rats [1,13,34,37,47,48]. Furthermore, CP administration increased hepatic NO which is a direct consequence of the up-regulated iNOS. NO can react with superoxide anions to form peroxynitrite (ONOO⁻), a potent biological oxidant that impairs mitochondrial and cellular functions, increases ROS production, and triggers DNA breaks by modifying the purine and pyrimidine bases [49]. Accordingly, our results showed a remarkable increase in DNA damage following CP administration. Gal supplementation effectively prevented CP-mediated increase in ROS, LPO, NO, and DNA damage, and boosted GSH and antioxidant enzymes. Thus, attenuation of oxidative/nitrative stress and restoration of antioxidant defenses represent a central part of the hepatoprotective mechanism of Gal. In accordance with these findings, Gal mitigated oxidative stress and alleviated cellular antioxidants in liver of rodent models of I/R injury [24] and CCl_4_ hepatotoxicity [23]. Gal possesses antioxidant and free-radical scavenging efficacies which could be attributed to the combination of 2,3 double bond with the 3-OH and 4-keto groups in its structure [50]. 

Sustained ROS/reactive nitrogen species (RNS) generation can activate stress signaling and pro-inflammatory pathways. NF-κB signaling is the major signal transduction pathway implicated in the gene regulation and activation of pro-inflammatory cytokines, including COX-2, iNOS, IL-1β, and TNF-α [34,51]. In liver diseases, increased pro-inflammatory chemokines and cytokines release from activated Kupffer cells leads to the recruitment of neutrophils and other inflammatory cells and activation of endothelial cells, resulting in more ROS/RNS production and in the development of liver necrosis [52]. Activated NF-κB signaling and increased inflammatory cells infiltration have been associated with CP-induced hepatic oxidative stress [1,13,37]. In this study, CP activated NF-κB, up-regulated COX-2, iNOS, IL-1β, and TNF-α, and increased serum IL-1β and TNF-α. Increased iNOS expression explained the high levels of NO observed in the liver of CP-intoxicated rats. ROS and pro-inflammatory cytokines are the two main factors inducing cell death. Here, CP induced apoptotic cell death as showed by increased BAX and caspase-3 and decreased BCL-2 which coincided with previous studies [1,5,53]. Accumulating evidence indicated that ROS and inflammatory cascade activation induce apoptotic cell death in the liver [1,4,9]. Herein, apoptosis is thought to be triggered by CP-mediated excessive ROS/RNS levels which in turn provoke DNA damage, eventually culminating in activation of mitochondrial apoptotic pathway through declined anti-apoptotic proteins. Moreover, ROS generated within the mitochondria during CP metabolism may lead to mitochondrial LPO, resulting in loss of mitochondrial membrane potential and cytochrome *c* release which eventually leads to hepatocytes apoptosis through caspase-3 activation. Therefore, suppression of CP-mediated ROS generation can protect against inflammation and apoptosis. This notion is supported by several studies demonstrated the protective effects of antioxidants against CP-induced hepatocyte apoptosis [1,4,13,34,37].

Gal suppressed NF-κB and its regulated pro-inflammatory mediators, BAX and caspase-3, and enhanced BCL-2 in liver of CP-induced rats. In the same context, Gal prevented apoptosis in I/R-induced liver injury [24] and cisplatin-induced nephrotoxicity [21] in rodents and suppressed NF-κB and apoptosis in human fibroblast-like synovium cells [54]. These studies along with our findings highlighted the potent anti-inflammatory and anti-apoptotic efficacies of Gal which were a direct consequence of attenuation of ROS generation. In addition to its radical-scavenging activity [50], we aimed to explore the mechanism underlying the antioxidant efficacy of Gal. We evaluated the role of Nrf2/ARE/HO-1 signaling in mediating the beneficial effect of Gal. Nrf2 suppresses oxidative stress by activating the expression of several antioxidant enzymes in response to ROS [9]. Therefore, Nrf2 activation is an effective strategy to combat CP-mediated oxidative stress. Here, CP suppressed Nrf2 signaling as shown by the decreased Nrf2, NQO-1, and HO-1. In a rat model of CP hepatotoxicity, we have previously reported declined hepatic Nrf2 and HO-1 expression [1]. Although ROS represent the induction signal of Nrf2 to dissociate from Keap1 and elicit the transcription of antioxidant genes, it suppressed Nrf2 signaling following CP injection. The declined Nrf2/HO-1 pathway could be a result of sustained surplus ROS levels which have been reported to suppress Nrf2 in liver [1,15,55,56], kidney [10,57], and endothelial cells [11].

Treatment with Gal activated hepatic Nrf2 signaling and consequently suppressed ROS and oxidative damage in CP-intoxicated rats. These results supported a recent study showed the ability of Gal to protect human keratinocytes against ultraviolet B-induced oxidative damage via up-regulating Nrf2 [25]. Nrf2 signaling might also have a key role in suppressing inflammation and apoptosis in CP-intoxicated rats pre-treated with Gal. Nrf2 can attenuate inflammation via suppressing NF-ĸB and pro-inflammatory cytokines [58]. This assumption was supported by studies showing increased severity of drug-induced hepatotoxicity in mice lacking Nrf2 [12], and activated NF-κB and inflammatory cytokines production in mouse primary astrocytes with Nrf2 knockout [59]. Recently, Gal has been shown to down-regulate NF-κB, pro-inflammatory cytokines, and iNOS and up-regulate IL-10 in lipopolysaccharide-stimulated microglia through Nrf2 activation [60]. 

The antioxidant and anti-inflammatory potential of Gal could also be connected to its ability to increase hepatic PPARγ expression in CP-intoxicated rats. In this context, multiple reports have focused on the involvement of PPARγ in the protection against drug-induced liver injury [9,13,34,51,61,62]. The molecular mechanism by which PPARγ exerts anti-inflammatory effects is the inhibition of NF-κB-dependent inflammatory genes transcription. PPARγ inhibits NF-κB through the reduction of p65 nuclear translocation and binding to DNA, inhibition of IκBα degradation [63], and NF-κB-dependent transcriptional control [64]. In addition, PPARγ activation can suppress NADPH oxidase-dependent ROS generation [65] and directly induce the expression of antioxidant enzymes [18]. Activation of PPARγ is therefore an important approach for the prevention of CP-induced hepatotoxicity. Herein, we found that Gal increased hepatic PPARγ expression in CP-induced rats. Consistently, a recent study has demonstrated that PPARγ mediated the anti-inflammatory effect of Gal in neuroinflammation [66]. In a viral mimic dsRNA analog-induced microglial cells, Gal inhibited the expression of pro-inflammatory mediators and inactivated NF-κB via PPARγ [66]. Moreover, several reports have strongly suggested a positive feedback loop between PPARγ and Nrf2 signaling and this maintains the expression of both transcription factors and their target genes in a simultaneous manner [67,68]. Additionally, we have reported that co-stimulation of Nrf2 and PPARγ can attenuate drug-induced toxicity [1,13,15,61,69].

## 5. Conclusions

**Figure 10:**
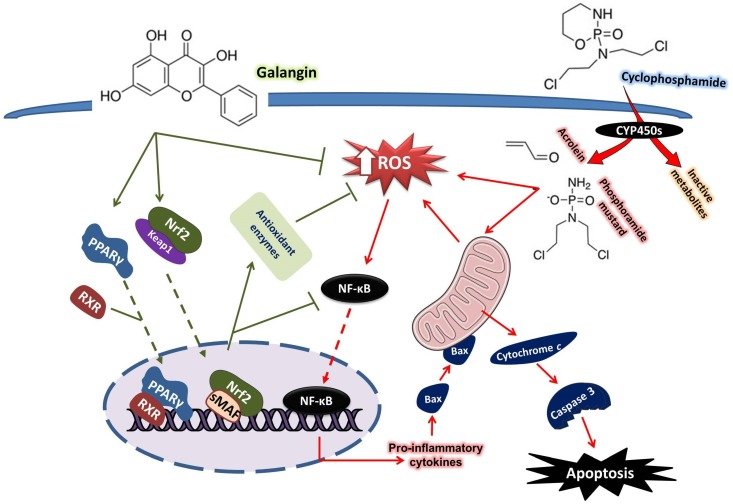
A schematic diagram illustrating the protective effect of galangin against cyclophosphamide hepatotoxicity. Galangin up-regulated Nrf2/HO-1 signaling and PPARγ, enhanced antioxidants, and suppressed ROS production, inflammation, and apoptosis in CP-induced rats. RXR, retinoid X receptor; CYP450, cytochrome P450; sMAF, small MAF proteins.

These findings provide in vivo evidence of the protective effect of Gal against CP hepatotoxicity. Gal attenuated CP-induced ROS production, LPO, DNA damage, inflammation, and apoptosis. Gal enhanced antioxidant defenses, activated Nrf2/HO-1 signaling, and up-regulated PPARγ in the liver of rats challenged with CP (summarized mechanistic pathways are represented in Figure 10). This study confers new information that the dual activation of Nrf2/HO-1 signaling and PPARγ mediated, at least in part, the hepatoprotective effects of Gal. Therefore, Gal might be supplemented as an adjuvant during chemotherapy to prevent hepatotoxicity. However, further basic and clinical investigations are needed to determine the exact mechanism underlying the hepatoprotective efficacy of Gal.

## Figures and Tables

**Table 1:** Primers used for qRT-PCR.

Gene	Forward Primer Sequence (5′-3′)	Reverse Primer Sequence (5′-3′)
*BAX*	AGGACGCATCCACCAAGAAG	CAGTTGAAGTTGCCGTCTGC
*BCL2*	ACTCTTCAGGGATGGGGTGA	TGACATCTCCCTGTTGACGC
*Casp3*	GAGCTTGGAACGCGAAGAAA	TAACCGGGTGCGGTAGAGTA
*TNFa*	AAATGGGCTCCCTCTCATCAGTTC	TCTGCTTGGTGGTTTGCTACGAC
*IL1b*	GACTTCACCATGGAACCCGT	GGAGACTGCCCATTCTCGAC
*NOS2*	ATTCCCAGCCCAACAACACA	GCAGCTTGTCCAGGGATTCT
*COX2*	TGATCTACCCTCCCCACGTC	ACACACTCTGTTGTGCTCCC
*NRF2*	TTGTAGATGACCATGAGTCGC	TGTCCTGCTGTATGCTGCTT
*NQO1*	GGCCATCATTTGGGCAAGTC	TCCTTGTGGAACAAAGGCGA
*HO-1*	GTAAATGCAGTGTTGGCCCC	ATGTGCCAGGCATCTCCTTC
*PPARg*	GGACGCTGAAGAAGAGACCTG	CCGGGTCCTGTCTGAGTATG
*Cyp2b1*	AGGACCATGGAGCCCAGTAT	GAGGTCCTGGTGGGAAGTTG
*Cyp2b2*	GTCCTGCATGGATGAGAGAGG	ATCATCAAGGGATGGTGGCCT
*Cyp2e1*	GCGGAGGTTTTCCCTAAGCA	GCGCAGCCAATCAGAAATGT
*Cyp2c11*	GGTCCAACACCTCTCCCAAT	CAAAGGGCTTCATGCCCAAA
*Cyp3a2*	GGAGTTGGCAAGGTCTGTGA	GATGTGGATGGAGATGGTCCC
*Actb*	AGGAGTACGATGAGTCCGGC	CGCAGCTCAGTAACAGTCCG

**Table 2:** Histopathological alterations in liver of control and CP-induced rats treated with galangin (Gal).

	Control	60 mg/kg Gal	CP			
			0 mg/kg Gal	15 mg/kg Gal	30 mg/kg Gal	60 mg/kg Gal
**Cytoplasmic vacuolations**	-	-	+++	+	+	+
**Congestion**	-	-	+++	+	+	-
**Hemorrhage**	-	-	++	+	+	-
**Fatty changes**	-	-	+	-	-	-
**Leukocyte infiltration**	-	-	++	+	-	-
